# Cross-Species Comparison of Genes Related to Nutrient Sensing Mechanisms Expressed along the Intestine

**DOI:** 10.1371/journal.pone.0107531

**Published:** 2014-09-12

**Authors:** Nikkie van der Wielen, Mark van Avesaat, Nicole J. W. de Wit, Jack T. W. E. Vogels, Freddy Troost, Ad Masclee, Sietse-Jan Koopmans, Jan van der Meulen, Mark V. Boekschoten, Michael Müller, Henk F. J. Hendriks, Renger F. Witkamp, Jocelijn Meijerink

**Affiliations:** 1 Top Institute Food and Nutrition, 9A, Wageningen, The Netherlands; 2 Division of Human Nutrition, Wageningen University, Wageningen, The Netherlands; 3 Division of Gastroenterology and Hepatology, Department of Internal Medicine, NUTRIM, Maastricht University Medical Center, Maastricht, The Netherlands; 4 Department of Animal Sciences, Wageningen University, Wageningen, The Netherlands; 5 Animal Sciences Group, Wageningen University and Research centre, Lelystad, The Netherlands; 6 Netherlands Organisation for Applied Scientific Research, TNO, Zeist, The Netherlands; University of Florida, United States of America

## Abstract

**Introduction:**

Intestinal chemosensory receptors and transporters are able to detect food-derived molecules and are involved in the modulation of gut hormone release. Gut hormones play an important role in the regulation of food intake and the control of gastrointestinal functioning. This mechanism is often referred to as “nutrient sensing”. Knowledge of the distribution of chemosensors along the intestinal tract is important to gain insight in nutrient detection and sensing, both pivotal processes for the regulation of food intake. However, most knowledge is derived from rodents, whereas studies in man and pig are limited, and cross-species comparisons are lacking.

**Aim:**

To characterize and compare intestinal expression patterns of genes related to nutrient sensing in mice, pigs and humans.

**Methods:**

Mucosal biopsy samples taken at six locations in human intestine (n = 40) were analyzed by qPCR. Intestinal scrapings from 14 locations in pigs (n = 6) and from 10 locations in mice (n = 4) were analyzed by qPCR and microarray, respectively. The gene expression of glucagon, cholecystokinin, peptide YY, glucagon-like peptide-1 receptor, taste receptor T1R3, sodium/glucose cotransporter, peptide transporter-1, GPR120, taste receptor T1R1, GPR119 and GPR93 was investigated. Partial least squares (PLS) modeling was used to compare the intestinal expression pattern between the three species.

**Results and conclusion:**

The studied genes were found to display specific expression patterns along the intestinal tract. PLS analysis showed a high similarity between human, pig and mouse in the expression of genes related to nutrient sensing in the distal ileum, and between human and pig in the colon. The gene expression pattern was most deviating between the species in the proximal intestine. Our results give new insights in interspecies similarities and provide new leads for translational research and models aiming to modulate food intake processes in man.

## Introduction

Various chemosensory mechanisms along the entire gastrointestinal tract are continuously monitoring the concentration of nutrients, digestion products and microbial metabolites. These chemosensory processes together with their effect on gastrointestinal hormone secretion are often referred to as “nutrient sensing”. The chemosensory mechanisms involve the action of different receptors and transporters generally located on membranes or within the cytoplasm of enterocytes, brush cells and enteroendocrine cells [1]. The latter cell types comprise about 1% of the epithelial cells in the intestine [2]. Nutrient sensing plays a pivotal role in the local and central regulation of food intake and gastrointestinal motility, secretion of mucus and enzymes, transport and uptake mechanisms [3]. According to the most common view, stimulation of G-protein coupled receptors (GPCRs) and/or ion-dependent nutrient transporters located at enteroendocrine cells, modulate the release of gut hormones like glucagon-like peptide-1 (GLP-1), cholecystokinin (CCK) and peptide YY (PYY) [1], [4]. For example, activation of the umami taste receptor (T1R1 and T1R3) by amino acids has been suggested to induce CCK secretion [5], whereas G-protein coupled receptor 120 (GPR120) responds to fatty acids, thereby stimulating GLP-1 and CCK secretion [6], [7]. In addition to receptors, several transporters for nutrients are involved in the modulation of gut hormone secretion. The sodium-glucose cotransporter member 1 (SGLT-1) has been suggested to induce GLP-1 secretion [8], [9]. Recently, the peptide transporter (PepT1) was also shown to stimulate GLP-1 secretion [10]. Secreted gut hormones can act via their corresponding receptors on vagal nerve afferents or via the endocrine pathway to affect food intake behavior [11]. The small intestine plays a prominent role in generating this feedback to the brain during and in between meals [12].

In spite of the importance of chemosensors in relation to food intake, there are only few studies describing the distribution of various chemosensors along the human intestinal tract. More knowledge on this (regional) distribution can provide better insight in the underlying nutrient-sensing mechanisms potentially involved in individual differences in food intake and the likeliness to develop metabolic diseases. The issue of cross-species comparison is important since the vast majority of studies in this field has been performed in rodents, such as the mouse [10], [13]–[15]. Pigs may serve as a more suitable animal model because pigs and humans show more similarity in gut physiology than mice and humans. Pigs are omnivorous and show a meal-eating pattern in their eating behavior. They have a comparable gastrointestinal physiology and intestinal transit time to humans [16]–[18]. However, despite these gross similarities it is not known to what extent the two species are similar with respect to gut nutrient sensing.

In the present study we extensively characterized the distribution of a number of receptors, transporters and hormones known to be involved in nutrient sensing focusing on the small intestinal tract of three species; pig, mouse and man. Next to measuring the expression of a selected set of genes involved in nutrient sensing we used Partial Least Squares (PLS) modeling to compare the three species. Lastly, the effect of fat/carbohydrate content in the diet on the expression of the selected genes was investigated.

## Methods

### Ethics statement

The use of human biopsy material for this study was approved by the Medical Ethical Committee of Maastricht University Medical Center+, the Netherlands (NCT02051881, NCT01574417). The porcine tissue was collected from control animals of a larger study, which was approved by the ASG-Lelystad Animal Care and Ethics Committee (Permit number: 2011135.c). Mice material was collected in a larger study which had been approved by the Local Committee for Care and Use of Laboratory Animals at Wageningen University (Permit number: 2010084.c).

### Tissue sampling

#### Human intestine

Biopsies were obtained from 40 healthy subjects (male and female between 21 and 82 years), who were referred for gastrointestinal endoscopy or participating as healthy controls in another study. Each subject gave written informed consent before participation. Exclusion criteria were as follows; the observation of any macroscopic or histologic abnormalities, history of severe cardiovascular, gastrointestinal/hepatic-, hematological/immunologic-, or metabolic/nutritional disease, major abdominal surgery interfering with gastrointestinal functioning or/and excessive alcohol consumption. All biopsies were taken with a standard forceps and the subjects were fasted prior to the endoscopic procedure. Due to the invasiveness of the procedure, it was only feasible to obtain biopsies from one or two locations in most subjects, except for the colon where mucosal tissue samples from three or four compartments were obtained. Duodenal tissue samples were taken from subjects who underwent an upper gastrointestinal endoscopy. These biopsies were taken at approximately 10 cm distal to the pyloric sphincter. Ileal and colonic biopsies were taken from subjects who underwent standard flexible colonoscopy. Ileal biopsies were taken at approximately 5 cm proximal to the ileoceacal valve. Colonic biopsies were taken from the ascending, transverse and descending colon and from the sigmoid colon, respectively. In nine subjects we were able to collect mucosal tissue samples at 40–45 cm distal to the pylorus, representing the proximal jejunum. All biopsies were snap frozen in liquid nitrogen and stored at −80°C until analysis.

#### Porcine intestine

To obtain tissue, six 10 week old male pigs (Large White x Landrace) were fasted overnight and killed by exsanguination under deep anesthesia. Immediately after this procedure, both the small and large intestine were excised and its total length was measured. From the small intestine, pieces of approximately 40 cm^2^ were cut out at 10 locations, namely at 3, 6, 20, 40, 50, 60, 70, 80, 90 and 98% of its total length (proximal to distal). These intestinal pieces were rinsed with water and scrapings were obtained. Scrapings were also taken from the cecum and at three locations in the large intestine, namely at 12.5, 37.5 and 75% of its total length. Apart from scrapings additional mucosal biopsies were taken from similar intestinal locations as mentioned above. Both the biopsies and scrapings were snap frozen in liquid nitrogen and stored at −80°C.

#### Mouse intestine

Male C57BL/6J mice (age 4 weeks) were housed 2 per cage in the light and temperature-controlled animal facility (12/12 (light/dark), 20°C) of Wageningen University. The mice had free access to water and received standard laboratory chow (RMH-B, Arie Blok BV, Woerden, the Netherlands) for 3 weeks, followed by a run-in period for 2 weeks during which 4 mice received chow diet and 8 mice received a 10E% low-fat diet. Subsequently, 4 mice remained on the chow diet, 4 mice remained on the low-fat diet and 4 mice received a 45E% high-fat diet for the experimental period of 2 weeks. The composition of the low-fat and high-fat diets has been previously described by de Wit et al. [19]. After the mice were fed, the small intestine of the sacrificed mice was excised. The small intestine was cut open longitudinally, divided in ten equal parts and scrapings were obtained. The colon was not sampled. These scrapings were snap frozen in liquid nitrogen and stored at −80°C until RNA isolation.

Both the biopsy and scraping sampling methods included similar mucosal and submucosal layers of the intestine. However, with biopsies a smaller surface of the intestine is taken in comparison to the scrapings. Therefore scrapings were expected to give a more representative determination of the epithelial gene expression than biopsies. Scrapings were taken from mice and pigs. However, to exclude the possibility that interspecies differences are caused by different sampling methods, gene expression profiles were compared for biopsies and scrapings in pigs. For five genes analyzed no differences were found, only CCK and PepT1 showing about 50% lower expression in biopsies compared to scrapings (results not shown).

### RNA isolation

RNA of the human and porcine samples was isolated by using TRIzol reagent (Life technologies, Bleiswijk, Netherlands) and further purified using the RNeasy mini kit (Qiagen) with on column DNase treatment (Qiagen, Venlo, Netherlands). The RNA isolation of the mouse scrapings was performed using the Promega SV total RNA isolation System (Promega Corporation, Madison, USA). RNA yield was measured with the Nanodrop ND-1000 Spectrophotometer and the quality of the human, mice and some porcine RNA samples was verified with an Agilent 2100 Bio analyzer (Agilent Technologies, Amstelveen, Netherlands).

### Quantitative PCR

Subsequently, 1 µg RNA was reversely transcribed using random primers with a Reverse Transcription System kit (Promega Corporation, Madison, USA) according to the manufacturer’s instructions. For the negative controls, the use of the enzyme reverse transcriptase (-RT control) was omitted.

The qPCR reactions were performed on the CFX384 Real-Time PCR Detection System (Bio-Rad Laboratories, Inc., Hercules, USA) using SensiMix SYBR No-ROX kit (Bioline, London, UK). Melt curve analysis and the amplification efficiency were used to verify the specificity of the amplification. Primers were designed using Beacon Designer 7.91 software, or primers were used from literature (Table S1 and S2). When using primers for Taqman analysis, the TaqMan Universal Master Mix II with UNG was used according to the manufacturer’s protocol. 36B4 (*RPLP0*) was used as reference gene to normalize the mRNA abundance of each gene [20].

Glucagon (*GCG*, as precursor for GLP-1), *CCK, PYY*, GLP-1 receptor (*GLP1R*), PepT1 (*SLC15A1*), SGLT-1 (*SLC5A1*), T1R3 (*Tas1R3*), GPR120 (*FFAR4*), T1R1 (*Tas1R1*), T1R2 (*Tas1R2*), GPR93 (*LPAR5*) and *GPR119* were measured in all human and pig samples. However, T1R2 could not be detected by qPCR in pig and human intestine (for both species 5 primers were tested), probably due to the low level of gene expression as also reported by others [21], [22]. Furthermore, despite the use of various primers T1R1 was still below detection level in the human samples and GPR119 was not detectable in the porcine samples. Lastly, GPR93 could not be quantified in pigs as the gene was not annotated. However, T1R1 was detected in mouse and pig, whereas GPR119 and GPR93 were demonstrated in mouse and man (Figure S5).

### Microarray hybridization and analysis

One hundred nanogram of RNA was used for Whole Transcript cDNA synthesis (Affymetrix, inc., Santa Clara, USA). Hybridization, washing and scanning of Affymetrix GeneChip Mouse Gene 1.1 ST arrays and Affymetrix GeneChip Porcine Gene 1.1 ST Arrays was carried out according to standard Affymetrix protocols. All arrays of the small intestine were hybridized in one experiment. Arrays were normalized using the Robust Multi-array Average method [23], [24]. Probe sets were assigned to unique gene identifiers, in this case Entrez IDs. The probes on the Mouse Gene 1.1 ST arrays represent 21,213 Entrez IDs. The probes on the porcine gene arrays represent 17,118 Entrez IDs [25]. Array data were analyzed using an in-house, on-line system [26]. All microarray data have been submitted to the Gene Expression Omnibus (GSE59054).

Both microarray and qPCR techniques have been extensively studied in the past decades and evidence for a strong correlation of the measured gene expression between qPCR and Microarray analysis has been assessed and proven in several papers [27]–[29]. Our own data were in accordance with these studies as comparisons of qPCR data with microarray data of 12 intestinal locations in pigs established that gene expresion patterns were highly similar when using both techniques (data not shown).

### Statistical analysis

Partial least squares (PLS) is a linear multidimensional fitting method. The method is used to relate sets of complex measurements X to a given external parameter Y. In this case the complex measurements are the measurement of gene expression and the external parameter is the location in the intestine for one of the species. The general formula for the method is Y  =  aX + b. Given an Y-vector and a X-matrix, PLS will calculate: a (loadings) and b (offset) which can then be used to predict the Y for any other set of data. The algorithm has many inbuilt features for scaling, filtering and cross validation (optimization of the number of factors used) of the data and is therefore very suited to be used with data where the relation between X and Y does not have to be directly linear. PLS was used to compare the intestinal expression patterns of eight genes between the species. Microarray data was used in log2 scale and subsequently all microarray and qPCR data were autoscaled to correct for the influence of the absolute intensities of the measurement. To prevent over fitting the PLS model was cross validated using a leave-one-out algorithm [30]. As the porcine data consisted out of a comprehensive map of the intestinal expression patterns for the selected genes, this dataset was selected to model the relation between gene expression levels and intestinal location. For this model the data of eight genes was used, as these were measured in all three species. Subsequently, the human and murine data were fitted into the porcine PLS model to compare the gene expression patterns for the different locations between the three species. PLS requires enough samples to cover the full range of the Y-values (locations in the intestine) to be fitted and enough samples to be able to cross validate the model. The sets used in this manuscript contain more than enough samples (pig 84 samples, human 63 samples, mouse 36 samples) to fulfill both these demands. For PLS analysis MATLAB (Version: 8.0.0.783, R2012b) and Winlin (version 1.8, TNO, Zeist, The Netherlands, [31]) were used.

## Results

### Comparison of the gene expression along the intestine between the three species

The relative gene expression pattern of each of the nutrient sensing related genes was measured at numerous intestinal locations in pig, man and mice. To compare the gene expression data of the three species, a PLS model was built for all three species. PLS analysis of the data gives a loading vector as listed in Table 1 and 2. In general, high positive loading vectors reflect high distal expression, while high negative loading vectors reflect high proximal expression. From the porcine loading vectors of Table 1 it can be seen that for example GPR120, PYY and glucagon give a positive contribution to the prediction of the location in the intestine i.e. in this model the samples at the distal intestine have relative higher expression of GPR120, PYY and glucagon than at the proximal intestine. The PLS model built on the human data, gave comparable results, with loading vectors of GPR120, PYY and glucagon being positive. To compare these two species with mouse, PLS models were built solely based on the small intestine. The loading vectors of these three species also show positive values for GPR120, PYY and glucagon and thus suggest a more distal role for GPR120, PYY and glucagon. Furthermore, the loading for CCK was negative in all three species, indicating that in all three species the relative expression of CCK is high in the proximal intestine, both in the small intestinal PLS models as well as in the complete PLS models.

**Table 1:** Loading vectors of the pig and human PLS model.

	Pig	Human
	5 factors	1 factor
**GPR120**	50.9005	6.1034
**Glucagon**	16.0706	1.8761
**PYY**	11.5365	10.035
**GLP-1R**	6.1094	−2.9701
**SGLT-1**	7.5340	−3.4999
**T1R3**	−0.2179	−15.0651
**PepT1**	−27.4127	−6.8932
**CCK**	−70.5	−5.534

Loading vectors obtained from PLS modeling of the complete intestinal data set of pig and human.

**Table 2:** Loading vectors of the pig, human and mouse PLS model of the small intestine.

	Pig	Human	Mouse
	6 factors	1 factor	3 factors
**GPR120**	89.5911	20.8624	7.1406
**Glucagon**	14.6987	3.129	9.0567
**T1R3**	9.3151	−1.1986	1.0031
**PYY**	4.6843	9.2374	3.7939
**SGLT-1**	1.2987	−1.3959	−13.1401
**GLP-1R**	0.1353	−1.1315	−9.6277
**PepT1**	−17.4913	−1.6997	6.533
**CCK**	−59.3902	−3.2496	−6.9791

Loading vectors obtained from PLS modeling of the small intestinal data set of pig, human and mouse.

**Figure 1:**
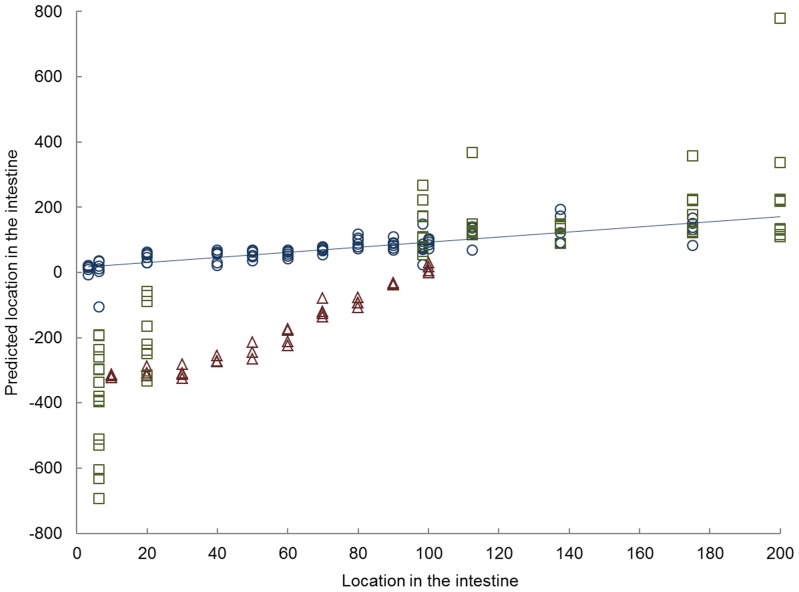
Partial least square analysis. Results of partial least squares (PLS) model in which porcine gene expression data (Ο) were used for regression analysis with locations in the intestine and the human (□) and murine data (Δ) were projected in the model. The PLS prediction model used 5 factors and has a R^2^ = 0.6541. The x-axis shows the location in the intestine, in which 0–100 resembles the small intestine from proximal to distal, 100–200 resembles the large intestine.

To further compare the gene expression data of the three species, the human en murine data were projected into the porcine model (5 factors, R^2^ = 0.6541) (Figure 1). For humans, the combined gene expression of all samples from distal ileum to colon were found to fit well to the porcine based model. The duodenal and jejunal samples, however, were more deviating from the modeled porcine samples when compared to the distal ileum and colon samples. Similar to the human proximal intestinal samples, the murine samples of the proximal small intestine are different from the modeled porcine samples. However, for the distal small intestine the difference between mice and pigs becomes less.

### Gene expression pattern along the intestine

**Figure 2:**
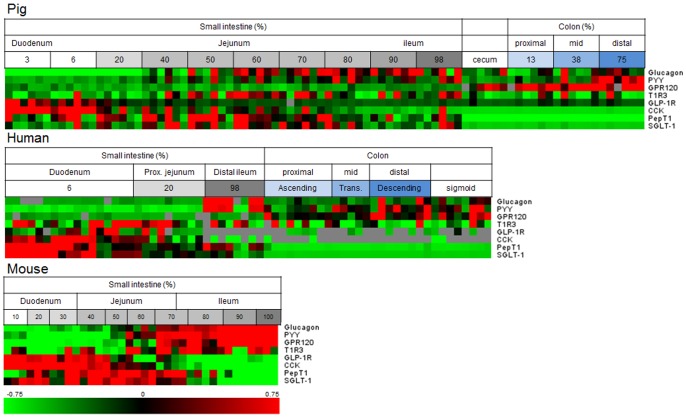
Heatmap of pig, human and murine gene expression results. Horizontally the individual samples of different parts of the intestine are aligned from proximal to distal and vertically the eight genes are shown. Green and red indicate low and high gene expression compared to average, respectively. Grey indicates samples that could not be analyzed/detected.

When studying the expression patterns for the nutrient sensing genes in more detail, some general expression patterns or specific patterns could be clearly observed for several of the genes (Figure 2). As shown in the heatmaps the gut hormones, glucagon (precursor for GLP-1), CCK and PYY and the receptor for GLP-1, all showed specific expression patterns along the intestine, which appeared similar for the three species.

Remarkably, the expression patterns of the nutrient transporters for di- and tri-peptides, PepT1, and for glucose, SGLT-1 were almost identical within each species. However, the expression patterns of both genes differed between the three species. In mice, GPR120 expression increased towards the distal small intestine, whereas in human and pigs the expression increased slightly along the small intestine. In human and pigs, the expression of this gene was more prominent in the colon.

Although the T1R family showed low to undetectable expression in the intestine, T1R3 was detected in all three species but showed a scattered expression pattern along the intestine.

Details for the expression patterns of each gene can be found in the supplemental data (Figure S1-5).

### Effect of diet on gene expression pattern in mice

**Figure 3:**
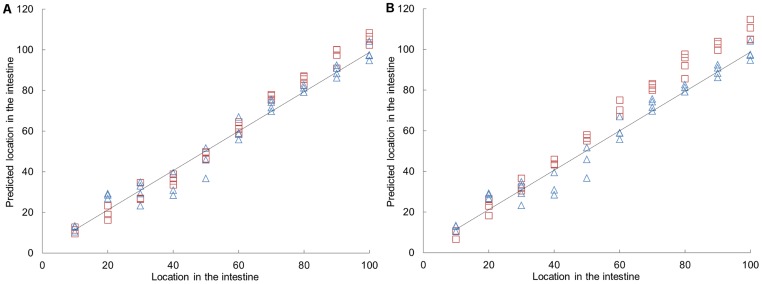
PLS prediction of locations along the intestine based on the gene expression in a sample. The PLS prediction model used 3 factors and has an R^2^ = 0.9681. The samples of mice fed a chow diet (Δ) were the basis of the model and the data of mice fed a low-fat (A, indicated with □) and high-fat diet (B, indicated with □) was fitted in the model.

To explore the effect of diet on expression of the studied genes, we also analyzed material from mice given different diets; chow, high fat-low carbohydrate or low fat-high carbohydrate diet. To analyze the effect of the three diets on the differences in gene expression of the eight genes along the small intestine, a PLS model was developed based on the data of the chow diet (using 3 factors, R^2^ = 0.9681). Subsequently, the results of the high-fat and low-fat diet were fitted in this model (Figure 3 and Figure S6). The model showed that location in the intestine had a greater effect on gene expression level than a dietary intervention. With respect to the selected eight genes, the low-fat diet did not show a high deviation from the chow diet. Expression after a high-fat diet, however, deviated slightly from expression after a chow diet, especially in the distal part of the small intestine.

## Discussion

Chemosensory receptors and transporters able to detect nutrients and other molecules present in the intestinal tract are pivotal for the regulation of food intake and other physiological responses to food ingestion. Moreover, nutrient sensing in the gut might also play a key role in maintaining metabolic homeostasis, for example of glucose. Impairment or changes of these nutrient sensing mechanisms may contribute to metabolic diseases, such as type 2 diabetes and obesity [1], [32], [33]. It is conceivable that a time- and site dependent interaction of food and digestion products with different chemosensory and other, including stretch and osmotic, sensors is key to these processes. However, detailed studies characterizing patterns of chemosensory receptors and transporters along the intestinal tract under normal physiological conditions are scarce. Moreover, information on interspecies differences is limited. Instead, the majority of studies focused on investigating a single gene in one or two species [6], [34]–[36].

Our data show a strong similarity between the expression of genes related to nutrient sensing in the distal ileum of the three species studied, which is mainly explained by the large contribution of glucagon and PYY to the model. Furthermore, the expression patterns in the colon of man and pig were highly comparable. Similarities in these locations of the intestine might be attributed to the similar high expression values of the GLP-1, PYY and GPR120 genes. Even though pigs have a higher relative volume and surface area of the large intestine than humans, we did not observe differences between the large intestine of pigs and humans as far as these genes are concerned [37]. As became clear from the loading vectors of all PLS models, GPR120, glucagon and PYY were predominantly expressed in the distal part of the intestine. GPR120 is expressed in L-cells of the intestine, which are enteroendocrine cells containing both PYY and GLP-1 [38]. To our knowledge the effect of GPR120 activation on PYY secretion has not been investigated yet, while a relation between GPR120 and secretion of GLP-1 and CCK has been described [6], [7], [39]. Hirasawa et al. showed that in both human and mouse intestine GPR120 was abundantly expressed especially in the colon [6]. This fatty acid receptor has been linked to obesity. In morbidly obese patients GPR120 expression in gastric tissue was higher compared to normal-weight individuals [40]. Moreover, a GPR120 mutation, found to be associated with obesity in man, influenced the ability to secrete GLP-1 in response to α-linolenic acid in enteroendocrine NCI-H716 cells [41].

When considering all genes combined, the most pronounced differences between the species studied here were found in the proximal small intestine. In the duodenum, the expression of the transporter genes SGLT-1 and PepT1 was deviating between the species (Figure 2 and S3). For PepT1, a higher gene expression in the human duodenum compared to ileum has been observed previously [42]. However, this is in contrast to findings of others who did not find significant differences between its expression in the duodenum and ileum [34]. In the porcine and murine intestine, the gene expression of PepT1 was highest in the jejunum, which is in agreement with findings of others [43], [44]. SGLT-1 gene expression along the intestine has been investigated in rodents, showing highest expression in the jejunum, whereas our results showed highest expression in duodenum and proximal jejunum [38], [45]. To our knowledge, SGLT-1 expression along the intestinal axis has not been reported previously for humans or pigs.

The basis that may underlie the different gene expression patterns in the proximal part of the intestine in the three species is unknown. However, gene expression of both transporters is known to be influenced by nutritional status or diet composition. High-protein diets are known to increase PepT1 mRNA expression and transporter activity [46], [47]. However, PepT1 increases found in these studies affected the middle and distal small intestine. Furthermore, a fed or fasted state might have influenced the amount of PepT1 mRNA, but studies show contradictory results [44], [48], [49]. Similarly, high-carbohydrate diets have been shown to increase SGLT-1 gene expression levels in the proximal and mid intestine but not in the distal small intestine [50], [51]. This increased expression is regulated by the sweet taste receptor [52], [53]. Therefore, it can be suggested that differences in dietary composition may contribute to the duodenal differences in expression patterns of these transporters in the three species. The high duodenal SGLT-1 expression in humans might be explained by a diet higher in carbohydrates compared to that of pigs and mice as the participants had no diet constrains. Interestingly, in our mice study the effect of a different fat content in the diet (at the expense of corn starch) on the gene expression of SGLT-1 was found to be much smaller than reported for effects of dietary carbohydrates in the literature. This could be due to the fact that in other studies sucrose was the main source of carbohydrates, whereas starch was the main dietary carbohydrate in the present study [53].

In spite of the fact that the expression patterns of the transporters, PepT1 and SGLT-1 along the intestine were found to differ between the species, Figure 2 and S3 show a striking and species-independent similarity in gene expression pattern between the two nutrient transporters. This might be due to a similar function in the intestine in the uptake of either peptides or glucose after the digestion of proteins and carbohydrates. These macronutrients are mainly digested by the action of pancreatic and brush border enzymes, which primarily takes place in the duodenum and proximal jejunum [54].

Due to the invasiveness of the procedure, the vast majority of duodenal, jejunal and ileal biopsies were obtained from different human subjects. Gene expression in the human duodenal samples showed a high inter-individual variation compared to the other regions of the intestine. This may at least in part be explained by different dietary habits between individuals. Additionally, genotypical differences might play a role as well.

Microarray and qPCR are two techniques for measuring gene expression and there is evidence for a strong correlation between qPCR and Microarray analysis [27]–[29]. However as the units of the output of both techniques are not directly comparable, the data needs further appropriate processing to make a reliable comparison of the data possible. PLS is a tool that can meet this demand. PLS is commonly used in the analysis of instrumental chemical measurements. Its use with biological data is increasingly being recognized [55], [56].

Our results show a high proximal expression of the GLP-1 receptor (Figure 2 and S2). This observation was remarkable as GLP-1 is mainly secreted in the distal parts of the intestine. However, it was recently shown that the GLP-1 receptor is expressed in both the small and large intestine [57]. In agreement with our data, that study showed that the vagal innervation of GLP-1 is reduced along the intestinal tract [57].

In contrast to T1R3, gene expression of its heterodimer T1R2 was not detected in both human and porcine intestine. A very low gene expression of T1R2 is consistent with findings from other studies [21], [22], [58]. An explanation for the much lower gene expression of T1R2 compared to the expression of T1R3 could be the potential dimerization of T1R3 with other GPRs [59]. This idea is supported by the fact that tissue explants of the jejunum and ileum from T1R3 knockout mice had no GLP-1 secretion compared to explants from wild type animals, whereas ileum explants of T1R2 knockout mice still secreted GLP-1. The authors of that study suggested that T1R3 can partially compensate for the loss of T1R2 [60]. T1R3 was expressed in the intestine of all three species suggesting a functional role in the intestine, possibly sensing of amino acids and/or sweet compounds.

In order to study the effect of fat content (at the expense of carbohydrate content) on expression of nutrient sensing related genes, we performed a two week diet intervention study in mice. The PLS model of these data showed slight differences in gene expression of the high-fat/low-carbohydrate diet compared to the chow diet in the distal region of the small intestine. Although it has been shown that a high-fat diet can induce changes in gene expression in several other pathways, like lipid metabolism and cell cycle, the nutrient sensing related genes studied here were hardly influenced by the fat/carbohydrate content in the diet [19], [61].

In conclusion, this study shows that the intestinal expression pattern of genes related to nutrient sensing show the highest similarity between humans, pigs and mice in the distal ileum and a high similarity between human and pigs in the colon. At the same time, more deviating gene expression patterns between the species were found for the proximal intestine. For the proximal small intestine some prudence in extrapolation of gene expression data from one species to the other may be required with respect to nutrient sensing. Lastly, we provided detailed information on the specific expression patterns of glucagon, CCK, PYY, GLP-1 receptor, PepT1, SGLT-1, T1R3 and GPR120 over the longitudinal intestinal axis of human, pigs and mice under normal physiological conditions. To our knowledge, this is the first study where gene expression of nutrient sensing related mechanisms has been characterized in such detail along the intestinal tract, and compared for relevant species, including human. Knowledge of the expression patterns of these nutrient sensing related genes in commonly used species may contribute to a better understanding of the satiating effects of specific diets and products. Furthermore, understanding their site- (and time-) specific interactions with molecular ligands may contribute to strategies for food intake modulation.

## Supporting Information

Figure S1 **Gene expression of glucagon and CCK along the intestine of human, pig and mouse.** Gene expression of glucagon in pig (A), human (B), mice (C) and gene expression of CCK in pig (D), human (E), mice (F) as assessed in numerous intestinal locations. Human and pig data show relative expression corrected for reference gene 36B4 determined using qPCR analysis. Mice results show microarray intensity.
(TIF)

Figure S2 **Gene expression of PYY and GLP-1 receptor along the intestine of human, pig and mouse.** Gene expression of PYY in pig (A), human (B), mice (C) and gene expression of GLP-1 receptor in pig (D), human (E), mice (F) as assessed in numerous intestinal locations. Human and pig data show relative expression corrected for reference gene 36B4 determined using qPCR analysis. Mice results show microarray intensity.
(TIF)

Figure S3 **Gene expression of PepT1 and SGLT-1 along the intestine of human, pig and mouse.** Gene expression of PepT1 in pig (A), human (B), mice (C) and gene expression of SGLT-1 in pig (D), human (E), mice (F) as assessed in numerous intestinal locations. Human and pig data show relative expression corrected for reference gene 36B4 determined using qPCR analysis. Mice results show microarray intensity. Both genes were highly expressed in all three species.
(TIF)

Figure S4 **Gene expression of T1R3 and GPR120 along the intestine of human, pig and mouse.** Gene expression of T1R3 in pig (A), human (B), mice (C) and gene expression of GPR120 in pig (D), human (E), mice (F) as assessed in numerous intestinal locations. Human and pig data show relative expression corrected for reference gene 36B4 determined using qPCR analysis. Mice results show microarray intensity.
(TIF)

Figure S5 **Gene expression along the intestine of human, pig and mouse.** Gene expression of T1R1 in pig (A), mice (D) and gene expression of GPR119 in human (B), mice (E) and gene expression of GPR93 in human (C), mice (F) as assessed in numerous intestinal locations. Human and pig data show relative expression corrected for reference gene 36B4 determined using qPCR analysis. Mice results show microarray intensity.
(TIF)

Figure S6 **Gene expression along the intestine of mice on chow, high-fat and low-fat diet.** Black bars show chow diet, grey bars show high fat diet and white bars show low fat diet. Results show mean microarray intensity of 4 mice per group and the standard deviation.
(TIF)

Table S1 **Porcine primers used for qPCR analysis.**
(DOCX)

Table S2 **Human primers used for qPCR analysis.**
(DOCX)
