# Supplementary figures and images for: Cross-Species Comparison of Genes Related to Nutrient Sensing Mechanisms Expressed along the Intestine
Source: PLoS One. 2014 Sep 12;9(9):e107531. doi: 10.1371/journal.pone.0107531 (PMC4162619; doi:10.1371/journal.pone.0107531)

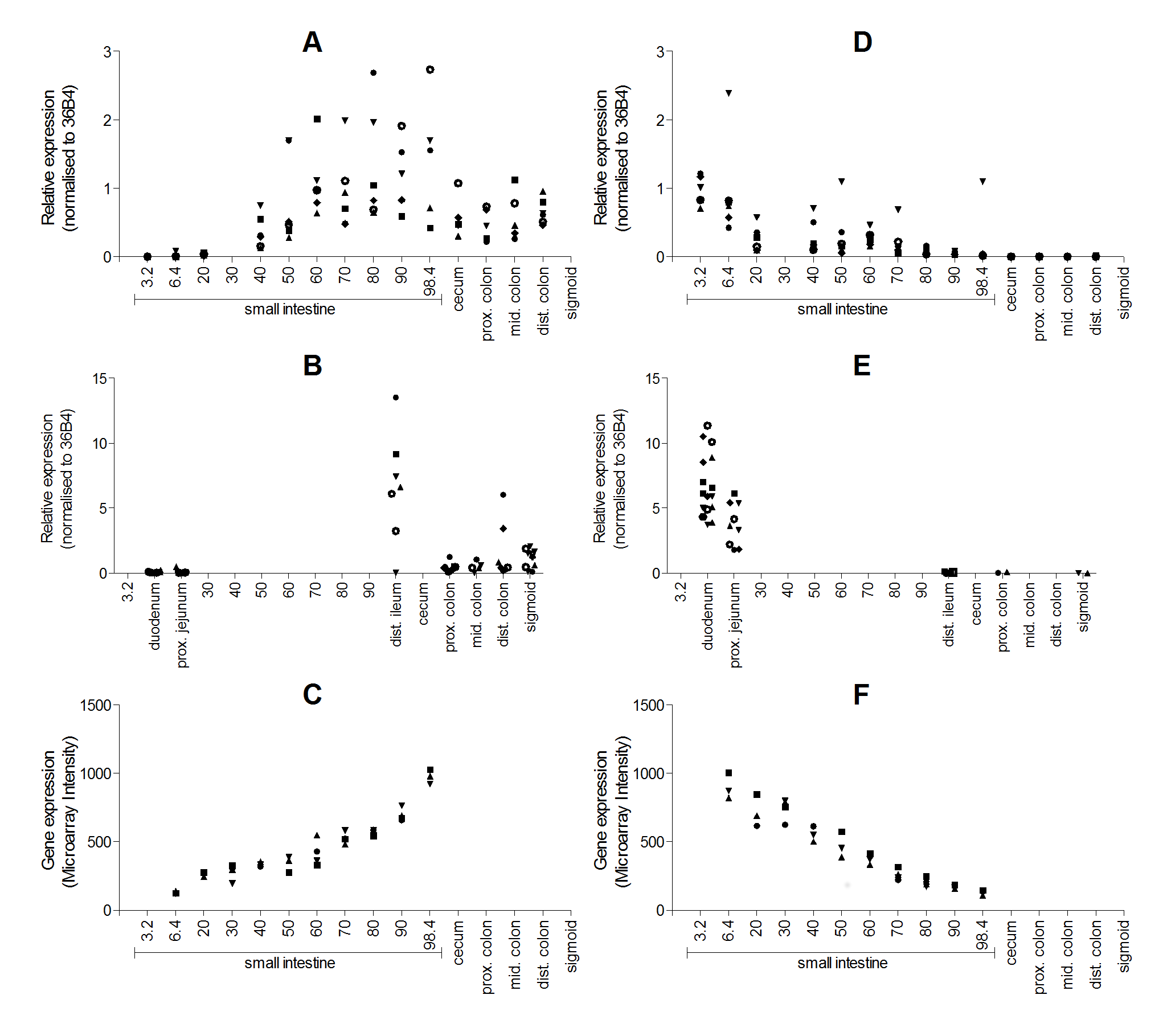

Supplement: Figure S1 — Gene expression of glucagon and CCK along the intestine of human, pig and mouse. Gene expression of glucagon in pig (A), human (B), mice (C) and gene expression of CCK in pig (D), human (E), mice (F) as assessed in numerous intestinal locations. Human and pig data show relative expression corrected for reference gene 36B4 determined using qPCR analysis. Mice results show microarray intensity. (TIF) [file pone.0107531.s001.tif]

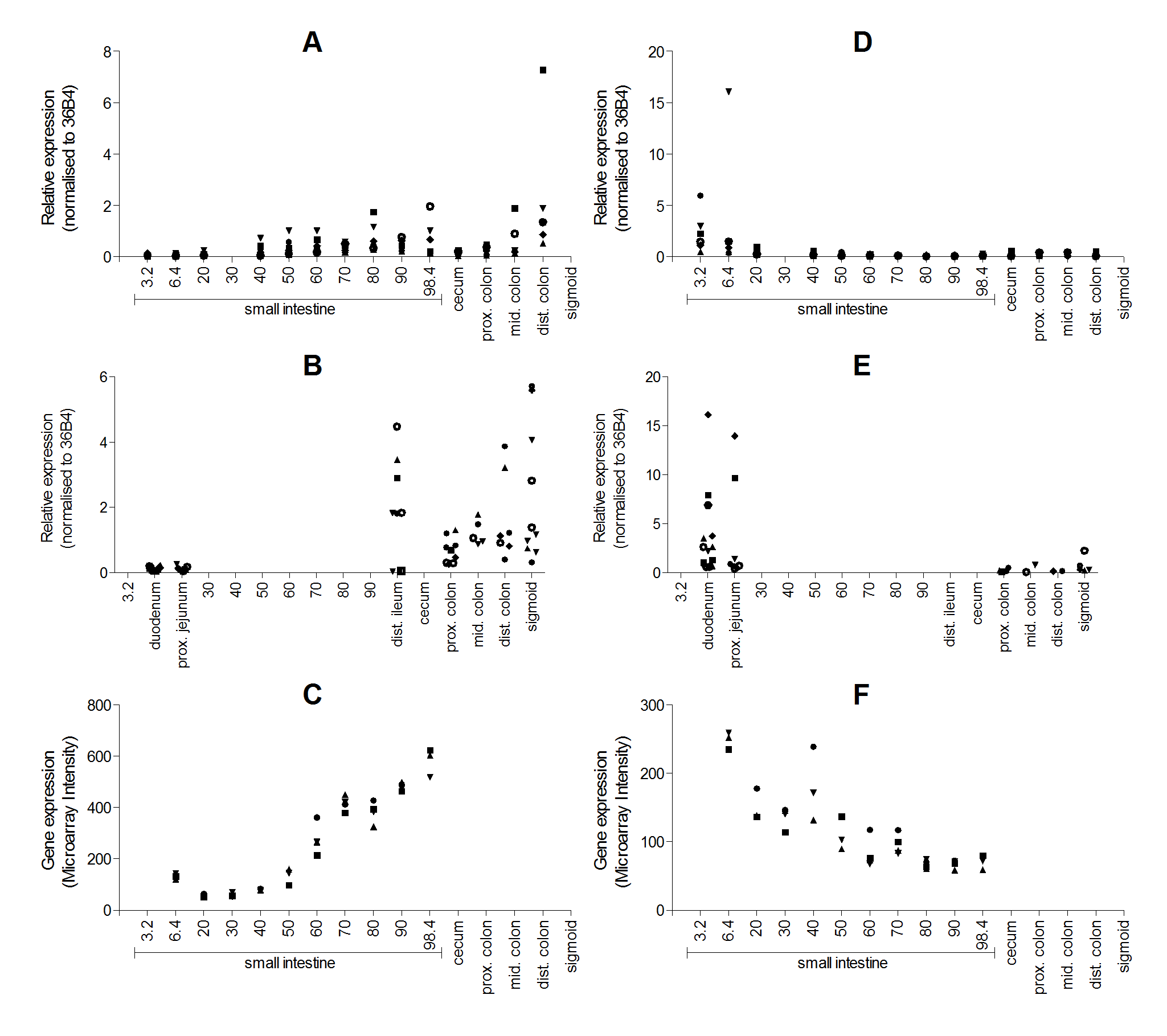

Supplement: Figure S2 — Gene expression of PYY and GLP-1 receptor along the intestine of human, pig and mouse. Gene expression of PYY in pig (A), human (B), mice (C) and gene expression of GLP-1 receptor in pig (D), human (E), mice (F) as assessed in numerous intestinal locations. Human and pig data show relative expression corrected for reference gene 36B4 determined using qPCR analysis. Mice results show microarray intensity. (TIF) [file pone.0107531.s002.tif]

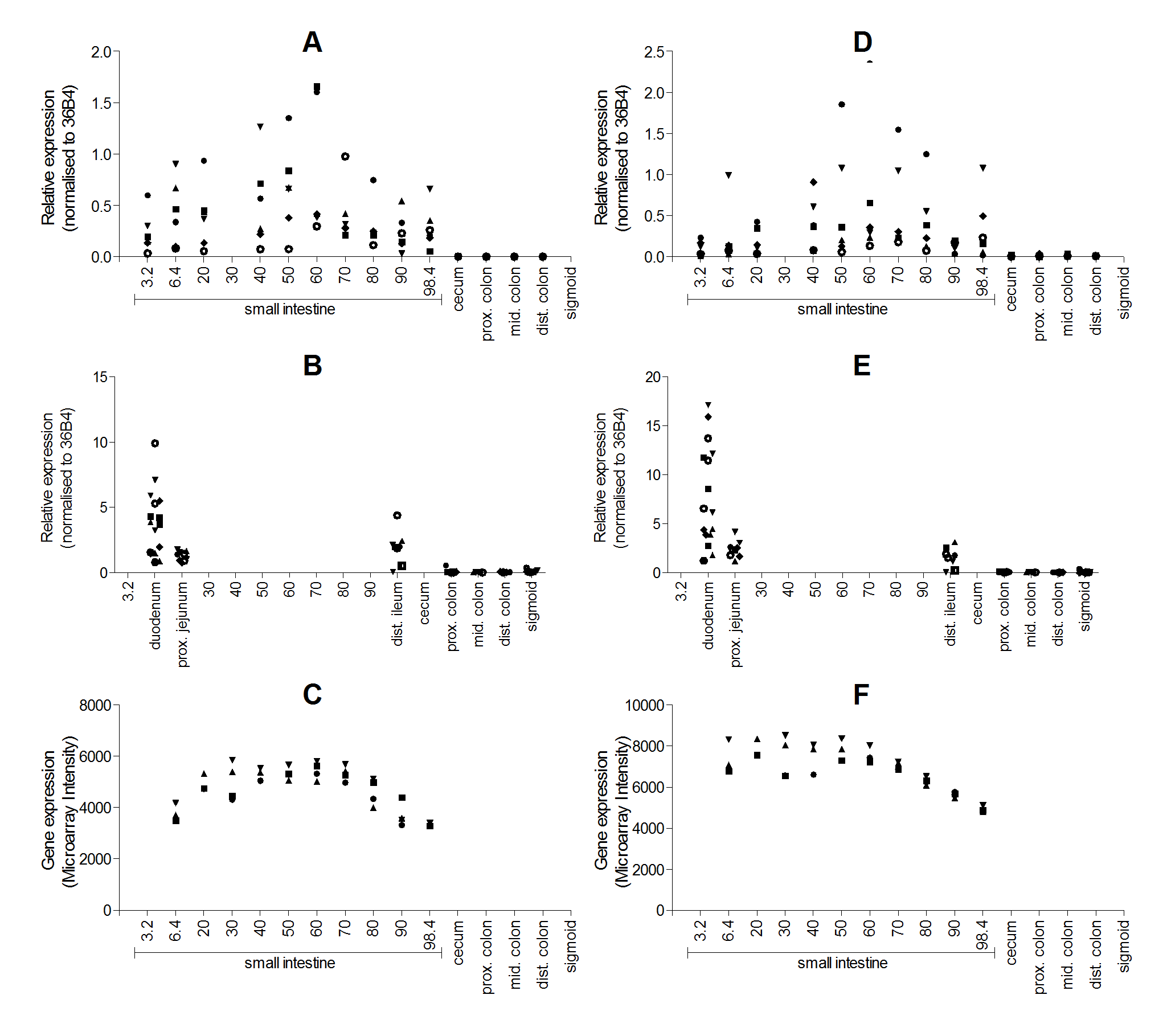

Supplement: Figure S3 — Gene expression of PepT1 and SGLT-1 along the intestine of human, pig and mouse. Gene expression of PepT1 in pig (A), human (B), mice (C) and gene expression of SGLT-1 in pig (D), human (E), mice (F) as assessed in numerous intestinal locations. Human and pig data show relative expression corrected for reference gene 36B4 determined using qPCR analysis. Mice results show microarray intensity. Both genes were highly expressed in all three species. (TIF) [file pone.0107531.s003.tif]

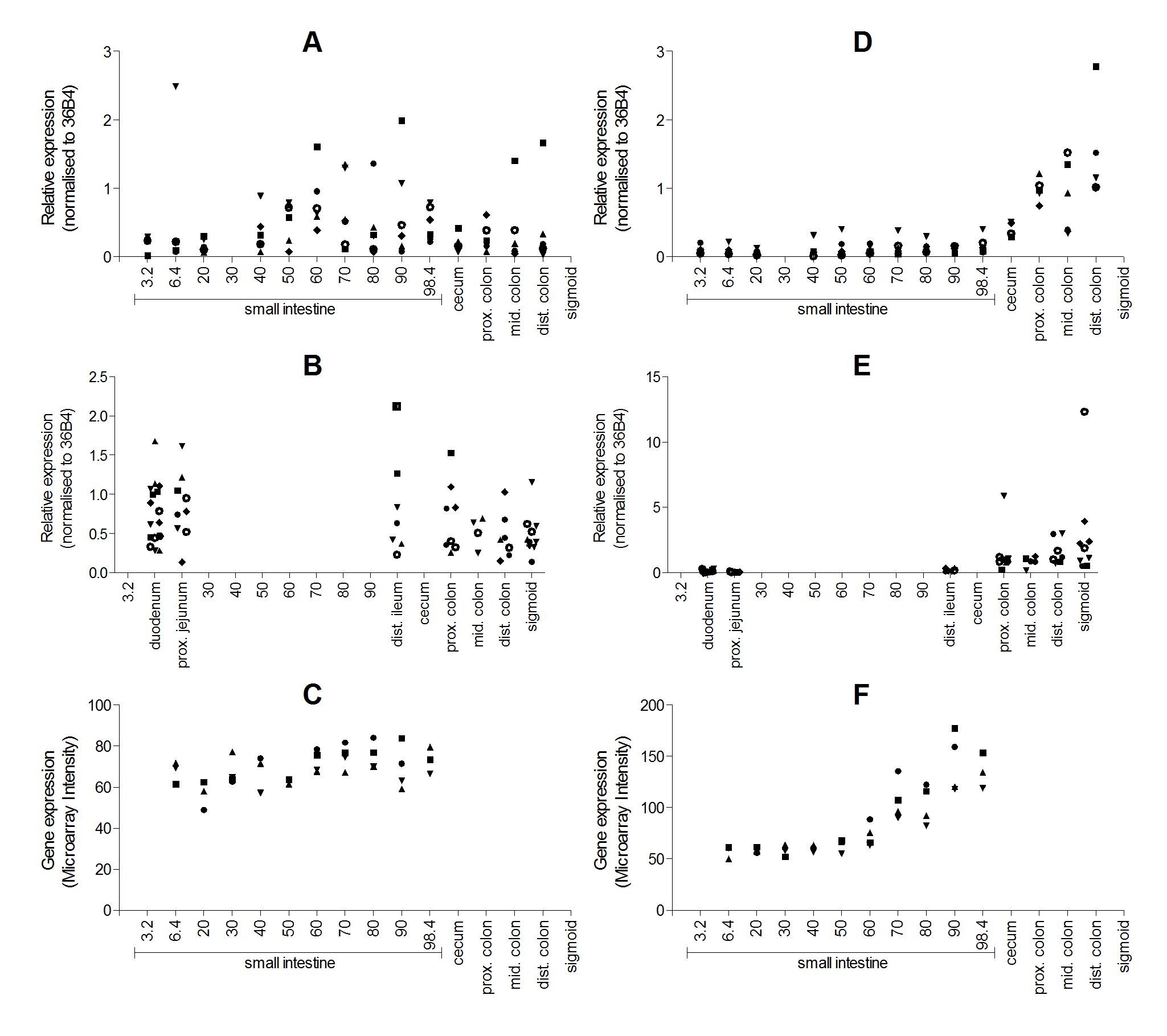

Supplement: Figure S4 — Gene expression of T1R3 and GPR120 along the intestine of human, pig and mouse. Gene expression of T1R3 in pig (A), human (B), mice (C) and gene expression of GPR120 in pig (D), human (E), mice (F) as assessed in numerous intestinal locations. Human and pig data show relative expression corrected for reference gene 36B4 determined using qPCR analysis. Mice results show microarray intensity. (TIF) [file pone.0107531.s004.tif]

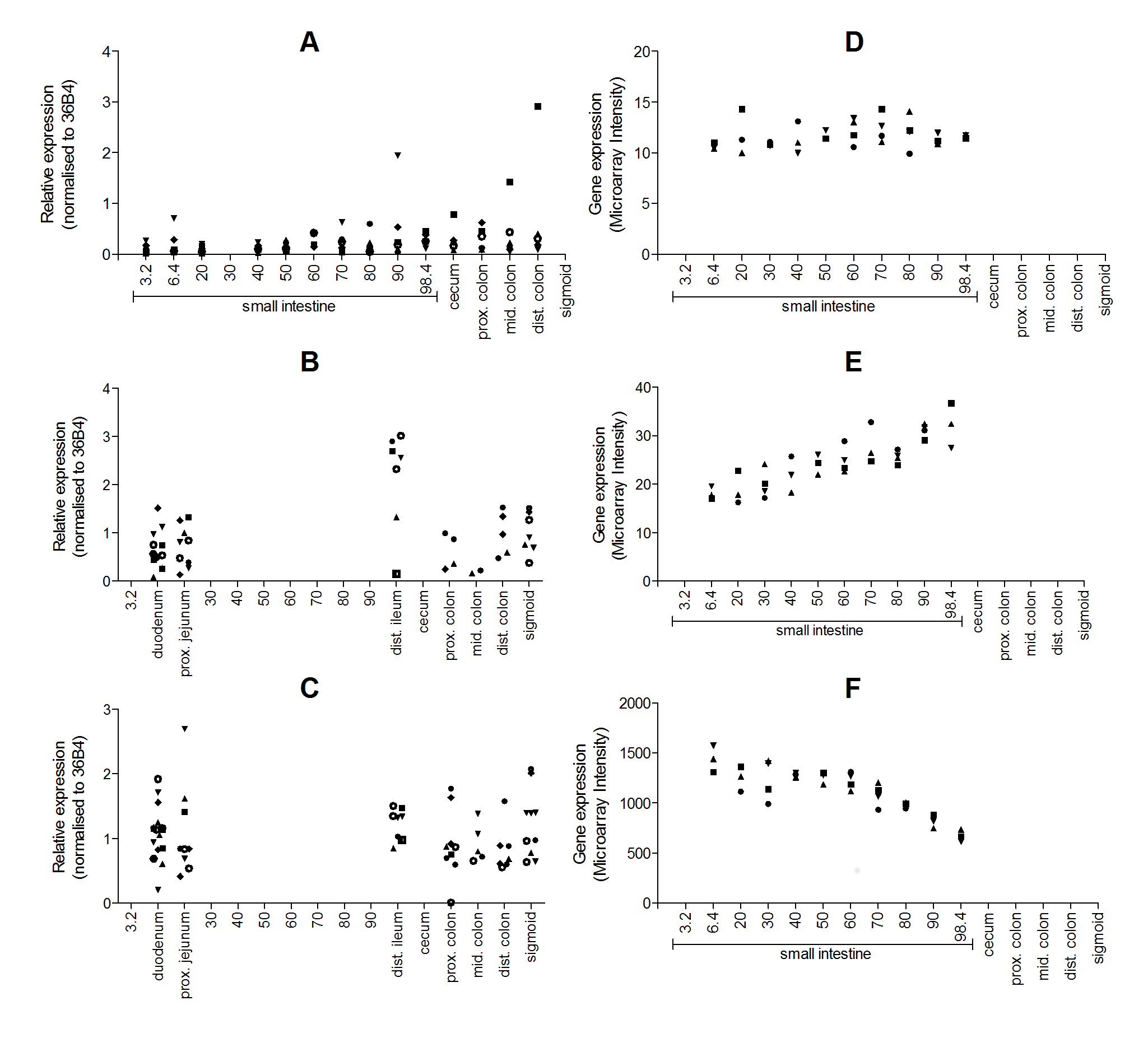

Supplement: Figure S5 — Gene expression along the intestine of human, pig and mouse. Gene expression of T1R1 in pig (A), mice (D) and gene expression of GPR119 in human (B), mice (E) and gene expression of GPR93 in human (C), mice (F) as assessed in numerous intestinal locations. Human and pig data show relative expression corrected for reference gene 36B4 determined using qPCR analysis. Mice results show microarray intensity. (TIF) [file pone.0107531.s005.tif]

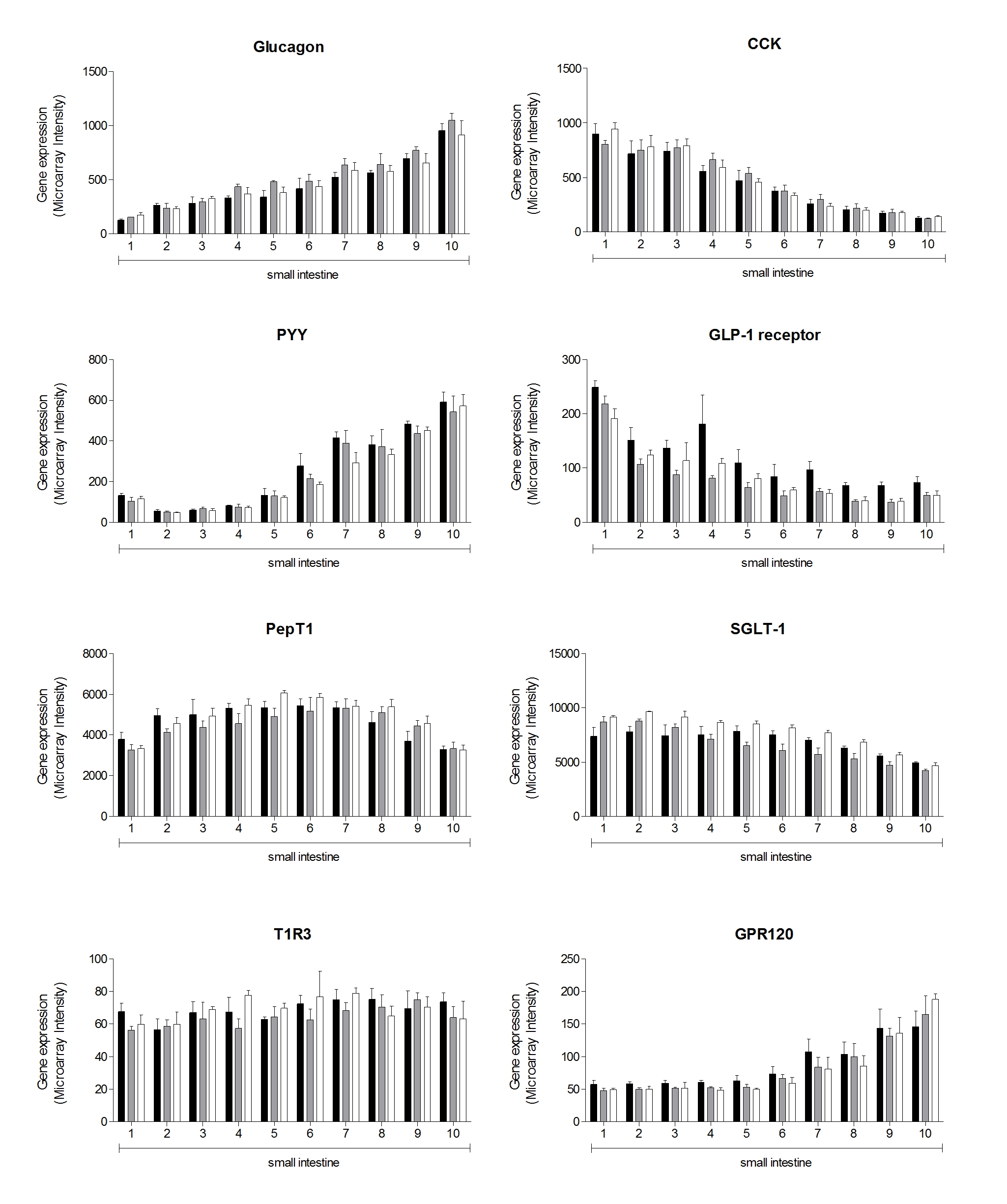

Supplement: Figure S6 — Gene expression along the intestine of mice on chow, high-fat and low-fat diet. Black bars show chow diet, grey bars show high fat diet and white bars show low fat diet. Results show mean microarray intensity of 4 mice per group and the standard deviation. (TIF) [file pone.0107531.s006.tif]
