# Supplementary material for: Cross-Species Comparison of Genes Related to Nutrient Sensing Mechanisms Expressed along the Intestine
Source: PLoS One. 2014 Sep 12;9(9):e107531. doi: 10.1371/journal.pone.0107531 (PMC4162619; doi:10.1371/journal.pone.0107531)
Supplement: Table S1 — Porcine primers used for qPCR analysis. (DOCX) [file pone.0107531.s007.docx]

| Name | Forward primer | Reverse primer | Reference sequence | Gene ID |
| --- | --- | --- | --- | --- |
| *GCG* | CAAGAGGAACAAGAATAACAT | AAGAACTTACATCACTGGTA | [NM_214324.1](http://www.ncbi.nlm.nih.gov/nuccore/NM_214324.1) | 397595 |
| *CCK* | TCAGAGGAGGCAGAAGAA | TGGACAATGTTACAGACAGATT | [NM_214237.2](http://www.ncbi.nlm.nih.gov/nuccore/NM_214237.2) | 397468 |
| *PYY* | AGATATGCTAATACACCGAT | CCAAACCCTTCTCAGATG | [NM_001256528.1](http://www.ncbi.nlm.nih.gov/nuccore/NM_001256528.1) | 100512433 |
| *GLP1R* | GCATCGTCAAGTACCTCT | GGATGATGAGCCAGTAGTTC | [NM_001256594.1](http://www.ncbi.nlm.nih.gov/nuccore/NM_001256594.1) | 100154059 |
| *Tas1R1* | ATCTGTTCTCGAGGCCAAGTCT | GCGAGTCCCCACTGTCACTAA | [XM_003356140.1](http://www.ncbi.nlm.nih.gov/nuccore/XM_003356140.1) | 100626842 |
| *Tas1R3* | GCTGGGCGACAGGACAG | TTGATTTCCTCCACAGCCAT | [NM_001113288.1](http://www.ncbi.nlm.nih.gov/nuccore/NM_001113288.1) | 100127440 |
| *SLC5A1* | AAAGGAGAGGTCTGGGATGGTAA | ATTTCCCTAGTGGCCTGAGATTG | NM_001164021.1 | 397113 |
| *SLC15A1* | AATTGTGTCGTTGTCCAT | AAGTCTGTGAGGTCATTG | [NM_214347.1](http://www.ncbi.nlm.nih.gov/nuccore/NM_214347.1) | 397624 |
| *LPAR5* | TGGGATGTGTCGTTTGTT | CCTTGATGCCTTGGTGAT | [NM_001204766.1](http://www.ncbi.nlm.nih.gov/nuccore/NM_001204766.1) | 100135678 |
| *RPLP0* | CTTTAGGCATCACCACTA | TGTCTCCAGTCTTAATCAG | [NM_001098598.1](http://www.ncbi.nlm.nih.gov/nuccore/NM_001098598.1) | 100049695 |
