# Supplementary material for: Cross-Species Comparison of Genes Related to Nutrient Sensing Mechanisms Expressed along the Intestine
Source: PLoS One. 2014 Sep 12;9(9):e107531. doi: 10.1371/journal.pone.0107531 (PMC4162619; doi:10.1371/journal.pone.0107531)
Supplement: Table S2 — Human primers used for qPCR analysis. (DOCX) [file pone.0107531.s008.docx]

| Name | Forward primer | Reverse primer | Reference Sequence | Gene ID |
| --- | --- | --- | --- | --- |
| *GCG* | AGCATTTACTTTGTGGCTGGAT | CGCTTGTCCTCGTTCATCTGAT | NM_002054.4 | 2641 |
| *CCK* | TATCGCAGAGAACGGATG | AGGTTCTTAACGATGGACAT | NM_000729.4, NM_001174138.1 | 885 |
| *PYY* | CTGAACCGCTACTACGCCTC | CGTCTCTTTTCCCATACCGCT | NM_004160.4 | 5697 |
| *GLP1R* | TTGGGGTGAACTTCCTCATC | CTTGGCAAGTCTGCATTTGA | NM_002062.3 | 2740 |
| *Tas1R3* | GCTAAATCACCACCAGAC | TGCACTGAAGAGTGTTGT | NM_152228.1 | 83756 |
| *SLC5A1* | TCTCTACCGTCTGTGTTG | GCTCTCCTGAAGATTCCT | NM_000343.3, NM_001256314.1 | 6523 |
| *SLC15A1* | CTTCTTCATCGTGGTCAA | GGTGGACAGGTTATCATC | NM_005073.3 | 6564 |
| *FFAR4* | CAGCAATCACATCTCCTACAT | CGTCCTGAATCGGTTCTAAG | [NM_001195755.1](http://www.ncbi.nlm.nih.gov/nuccore/NM_001195755.1), NM_181745.3 | 338557 |
| *GPR119* | Hs02825719_s1 (Life technologies) | | NM_178471.2 | 139760 |
| *LPAR5* | TTCTCGCATAGTGACTTGT | TTGGACTTGGATGTTGTTG | NM_001142961.1, NM_020400.5 | 57121 |
| *RPLP0* | CGGGAAGGCTGTGGTGCTG | GTGAACACAAAGCCCACATTCC | NM_001002.3, NM_053275.3 | 6175 |
